# Potassium channels in depression: emerging roles and potential targets

**DOI:** 10.1186/s13578-024-01319-0

**Published:** 2024-11-11

**Authors:** Jiahao Zhang, Yao Zhu, Meng Zhang, Jinglan Yan, Yuanjia Zheng, Lin Yao, Ziwei Li, Zihan Shao, Yongjun Chen

**Affiliations:** 1 Institute of Acupuncture and Moxibustion, Shandong University of Traditional Chinese Medicine, Jinan, 250355 China; 2 Shandong Key Laboratory of Innovation and Application Research in Basic Theory of Traditional Chinese Medicine, Shandong University of Traditional Chinese Medicine, Jinan, 250355 China; 3 Key Laboratory of Traditional Chinese Medicine Classical Theory, Ministry of Education, Shandong University of Traditional Chinese Medicine, Jinan, 250355 China; 4 Shandong Provincial Engineering Research Center for the Prevention and Treatment of Major Brain Diseases with Traditional Chinese Medicine, Shandong University of Traditional Chinese Medicine, Shandong University of Traditional Chinese Medicine, Jinan, 250355 China

**Keywords:** Depression, Potassium channels, Neuronal activity, SNP

## Abstract

Potassium ion channels play a fundamental role in regulating cell membrane repolarization, modulating the frequency and shape of action potentials, and maintaining the resting membrane potential. A growing number of studies have indicated that dysfunction in potassium channels associates with the pathogenesis and treatment of depression. However, the involvement of potassium channels in the onset and treatment of depression has not been thoroughly summarized. In this review, we performed a comprehensive analysis of the association between multiple potassium channels and their roles in depression, and compiles the SNP loci of potassium channels associated with depression, as well as antidepressant drugs that target these channels. We discussed the pivotal role of potassium channels in the treatment of depression, provide valuable insights into new therapeutic targets for antidepressant treatment and critical clues to future drug discovery.

## Introduction

Depression is a prevalent mood disorder that imposes considerable economic strain on both families and society [1]. Globally, more than 300 million individuals are affected by depression [2], yet upwards of 45% of patients do not experience a significant response to antidepressant medication treatments [3]. Despite its widespread occurrence, the etiology of depression is multifaceted, its manifestations are profound, and its pathogenesis remains enigmatic [4]. This underscores the critical need for continued investigation into its underlying mechanisms and the identification of novel therapeutic targets. Ion channels are protein structures located on cell membranes that permit specific ions, these include cation channels, such as potassium channels, sodium channels, and calcium channels, as well as anion channels such as chloride channels. Potassium channels significantly influence neural activity by regulating the duration of action potentials and neurotransmitter release [5–7]. Previous studies highlight that potassium channels are closely linked to the occurrence and progression of various diseases, such as arrhythmias [8], epilepsy [9], Alzheimer's disease [10], cerebral ischemia [11], and diabetes [12], etc. Intriguingly, an increasing number of recent studies indicate that changes in neural activity can contribute to the development of mental disorders, including depression [13–15]. Potassium channels, which are essential for neuronal excitability and the conduction of signals, play a pivotal role in modulating depressive states [16]. Therefore, understanding the relationship between ion channels and depression is crucial for elucidating pathogenesis and developing effective treatments for depression.

## Classification and functions of potassium channels

**Fig. 1:**
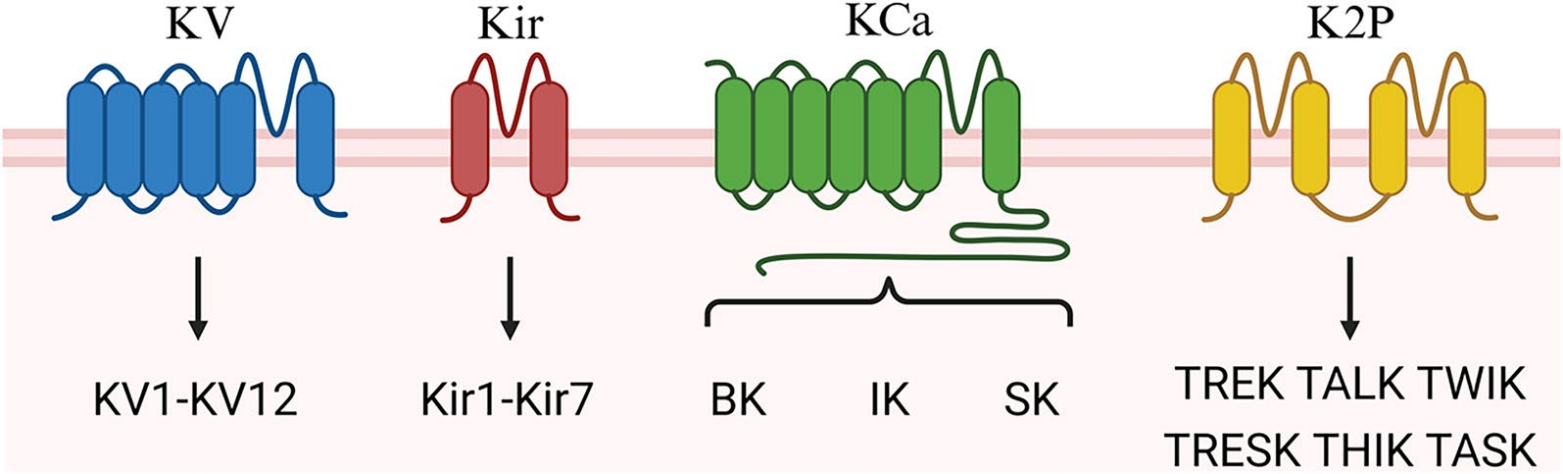
Structure and Classification of Potassium Channels. Based on subunit structure, potassium channels can be divided into four families: KV, Kir, KCa, and K2P

Potassium channels represent the most diverse subtype among all ion channels and are distributed in both excitatory and inhibitory neurons. They play essential roles in both excitable and non-excitable cells [17]. These channels consist of tetrameric integral membrane proteins that form transmembrane water pores. Based on the primary amino acid sequence of the pore-containing α-subunits as well as their physiological and pharmacological characteristics [18], potassium (K⁺) channels can be categorized into four main classes: (1) voltage-gated K^+^ channels (KV); (2) inward rectifier K^+^ channels (Kir); (3) calcium-activated K^+^ channels (KCa); and (4) two-pore domain K⁺ channels (K2P) (Fig. 1). They play an essential role in regulating activities such as heart rate, muscle contraction, neurotransmitter release, neuronal excitability, insulin secretion, electrolyte transport in epithelial cells, cell volume regulation, and apoptosis [19].

The KV family represents the most diverse and functionally complex group of potassium channels [20]. The KV channel structure consists of four subunits, each arranged around a central pore and containing six transmembrane helices (TMs) [21]. This family encompasses twelve distinct members, designated as KV1 to KV12 [22]. They regulate neuronal excitability by modulating cell membrane repolarization [23], influencing neuronal excitability and various electrophysiological properties such as spike membrane potential, action potential waveform, and frequency [24]. Most KV channels open during membrane depolarization and close during hyperpolarization, which helps return the cell to its resting state [25].

The primary structures of Kir channels feature a conserved motif comprising two putative membrane-spanning domains (TM1 and TM2), and they comprise the subfamilies Kir1 to Kir7 [26]. Kir channels allow K^+^ to move more easily into rather than out of the cell. The primary physiological roles of Kir channels are to stabilize the resting membrane potential (RMP) and regulate the excitability of excitable cells [27]. Kir channels coordinate both passive and active electrical properties of cells and participate in G protein-coupled receptor signaling, thereby linking cellular metabolism with membrane excitability [28].

KCa channels are a type of potassium channel whose activity is modulated by fluctuations in intracellular calcium ion concentration. KCa channels are classified into three primary categories: big conductance K + channels (BK), intermediate conductance K + channels (IK), and small conductance K + channels (SK) [29]. BK channels are composed of tetrameric structures, with each assembly consisting of four pore-forming α-subunits, each containing seven transmembrane domains (TMs). Similarly, IK and SK channels are also tetrameric, with each subunit exhibiting a Shaker-like membrane topology comprising six TMs [30]. These channels open or close in response to fluctuations in calcium levels, affecting the amplitude and duration of calcium transients. This modulation influences downstream signaling pathways triggered by changes in intracellular calcium concentration [31]. In central neurons, KCa channels regulate neuronal excitability, firing patterns, and synaptic signal transduction. They also contribute to action potential repolarization, mediate the rapid phase of afterhyperpolarization (AHP), and influence neurotransmitter release [32].

K2P channels are characterized by two pore-forming domains and four transmembrane domains. K2P channels can be subdivided into various types based on amino acid homology, including TREK, TALK, TWIK, TRESK, THIK and TASK [33]. They can be activated across the entire physiological voltage range and are crucial components of background potassium currents [34]. K2P channels are essential for neuronal excitability [35]. They play critical roles in regulating and maintaining the RMP, modulating cellular excitability, and balancing intracellular and extracellular potassium ions[36].

## Genetic links between potassium channels and depression

Extensive research has underscored the profound influence of genetic factors on the psychopathology of depression and other mental health disorders [37]. One such study examined frozen striatal samples from the Stanley Foundation Brain Collection, utilizing Reverse Transcription-Polymerase Chain Reaction (RT-PCR) techniques. The analysis included 60 brain samples, drawn from both individuals with depression and those without the disorder. The findings indicated a significant upregulation of the KCNJ4 and KCNJ1 subunits of the Kir2.3 potassium channel, as well as the KCNN3 subunit of the SK3 channel, in the striatal samples from individuals with major depression. In contrast, the expression of the KCNA1 subunit of the KV1.1 channel and the KCNS3 subunit of the KV9.3 channel was downregulated in the striatum of these subjects. These alterations in potassium channel gene expression within the striatum-nucleus accumbens region suggest a decrease in electrical activity in this brain area, which may be associated with the symptoms and therapeutic responses observed in depression [38].Single nucleotide polymorphisms (SNPs), a key form of genetic variation, have significant implications for genetic research and clinical diagnosis [39]. A study involving 449 patients with MDD and 421 healthy controls to examine the association between polymorphisms in the KCNK2 gene and MDD, as well as the efficacy of antidepressant treatment. They found that the genotype frequency of rs6686529 differed significantly between MDD patients and controls. Individuals with the homozygous genotype showed greater susceptibility to MDD compared to those with the heterozygous genotype, suggesting a potential dominant effect of this polymorphism on MDD [40]. Furthermore, this polymorphism affected the efficacy of antidepressant treatment: homozygous individuals were more likely to achieve remission after 8 weeks of treatment compared to G allele carriers. These findings indicate that KCNK2 is involved in both susceptibility to MDD and response to antidepressant therapy.

After investigating six SNPs in the KCNK2 gene among 590 patients with MDD and 441 controls, it was found that the A homozygote of rs10779646 was significantly more prevalent in patients than in controls. Additionally, the G allele of rs7549184 was associated with the presence of psychotic symptoms and disease severity [41]. A study examined SNPs in the KCNK2 gene in 565 patients with major depression and found that variations in SNPs (rs12136349, rs2841616, rs2841608) correlated with the response to citalopram treatment. This suggests that genetic variations in KCNK2 could help identify individuals at risk of treatment resistance [42].

Recent research indicates that hyperpolarization-activated cyclic nucleotide-gated (HCN) channels are essential for regulating neuronal excitability and are widely expressed in the brain. The HCN4 gene, which is expressed in brain regions linked to mood and depression, has two SNPs (rs3859014 and rs12905212) significantly associated with major depression [43]. A meta-analysis identified a novel SNP, rs79878474, located on chr11p15, which is likely associated with depression. The KV3 and Kir-6.2 potassium channels, encoded by the KCNC1, KCNJ11, and ABCC8 genes, show a close relationship with depression in children and adolescents [44]. Additionally, SNPs rs9394578 and rs2815095, associated with the KCNK5 gene, are significantly linked to depression [45]. Current research on potassium channel genes and depression primarily focuses on the TREK-1 type potassium channels (Table 1), with less extensive studies on other potassium channel types, underscoring the need for further investigation in this area.

**Table 1:** SNPs of Potassium Channels Associated with Depression

SNP	Gene	Properties	References
rs6686529	KCNK2	The occurrence rate of homozygotes is significantly higher in the patient group than in the control group	[40] [41]
rs7549184			
rs10779646			
rs12136349	KCNK2	Variants in KCNK2 are associated with treatment responses	[42]
rs2841616			
rs2841608			
rs3859014	HCN4	Expression of the HCN4 gene in brain regions is associated with emotions and depression	[43]
rs12905212			
rs79878474	KCNC1	The potassium channels KV3 and Kir-6.2 are closely associated with depression in children and adolescents	[44]
rs9394578	KCNK5	Genes are significantly enriched in severe depression	[45]
rs2815095			

## Pathological roles of potassium channels for depression

The pathogenesis of depression is complex. Current research suggests that the onset of depression is closely related to monoamine neurotransmitters [46], the neuroendocrine system [47], immune cytokines [48], neural plasticity [49], and gut microbiota [50], etc. However, these mechanisms and hypotheses do not fully explain the pathogenesis of depression. Increasing studies indicate that potassium channels, which are fundamental to neuronal excitability and signal transmission, are closely associated with both the onset and treatment of depression channels[51](Table 2).

**Table 2:** Functions of Different Subtypes of Potassium Channels and Their Roles in Depression

Channels	Types	Properties and functional relevance	Potassium channels in different brain regions	Activity changes in depression
KV	KV1	Increases the threshold of action potentials, reduces the width of action potentials, and inhibits neuronal excitability [81–83].	Hippocampal, NAc	Increases[76, 84]
	KV3	Achieves rapid repolarization of action potentials to enable high-frequency firing with temporal precision [85].	Hippocampal	Decreases[61, 64]
	KV4	Modulates the frequency and morphology of action potentials, prolonging the duration of action potentials during repetitive firing. [86–88].	NAc	Decreases[77, 89, 90]
	KV7	Slows the afterhyperpolarization and adjusted the RMP to stabilize neuronal excitability [91–93].	mPFC, hippocampal	Decreases[78, 94, 95]
Kir	Kir3.1 (GIRK)	Maintains RMP, cellular excitability, and modulating inhibitory neurotransmitters to preserve homeostasis and specific synaptic plasticities within the body. [96, 97].	hippocampal	Increases[98, 99]
	Kir4.1	Maintains high K^+^ conductivity in astrocytes and preserves RMP. [100, 101].	mPFC, hippocampal, LHb	Increases[57, 66, 79]
	Kir6.1 (K-ATP)	Leads to hyperpolarization of postsynaptic membranes and inhibition of neuronal activity, reducing neuronal excitability. [102].	Hippocampal	Increases[67, 68, 103]
KCa	BK	The opening of BK channels can repolarize the membrane and prevent Ca^2+^ entry into the cell [104].	mPFC	Decreases[59, 105]
	SK	An increase in intracellular Ca^2+^ following synaptic stimulation activates SK channels, which inhibits membrane excitability, promotes dendritic integration, and regulates the induction of synaptic plasticity [106] [107].	mPFC, VTA	Increase[58, 62, 80]
K2P	TREK-1	Maintaines the RMP near the potassium equilibrium potential to regulate cellular excitability. [108–110].	mPFC, hippocampal	Increases[69, 70]
	TASK-3	Regulates neuronal activity by influencing the RMP of neurons [111]/	Hippocampal	Increases[71] [112, 113]

### Potassium channels in the mPFC region

The pathogenesis of depression involve several brain regions, including the frontal cortex (PFC), hippocampus, nucleus accumbens (NAc), ventral tegmental area (VTA), lateral habenula (LHb) and dorsal raphe nucleus [52]. Among these regions, the PFC is essential for regulating emotions, cognition, and decision-making [53]. It plays a significant role in emotional regulation and social interaction [54], making PFC activity a predictor of treatment outcomes for depression [55]. In individuals with major depressive disorder (MDD), the protein expression of inward-rectifying Kir4.1 channels in the parietal cortex is notably increased [56]. Additionally, studies conducted in vitro, in vivo, and post-mortem have suggested that the heightened activity of Kir4.1 channels may contribute to the development of depression [57]. Moreover, the excitability of pyramidal neurons in the cingulate cortex is enhanced, as indicated by alterations in membrane potential and spike frequency in response to current injection, which is associated with increased activity of SK channels [58] and the inhibition of BK channels [59].

A growing body of evidence indicates that potassium channel activity in the medial prefrontal cortex (mPFC) is closely linked to the onset and treatment of depression. Research suggests that KV7/KCNQ/M currents modulate the excitability and synaptic transmission of neurons in the PFC of rats. The inhibition of muscarinic potassium currents has been shown to significantly enhance the amplitude of excitatory postsynaptic potentials and depolarize the resting membrane potential (RMP), thereby influencing the pathophysiology of depression [60]. Furthermore, other studies have revealed an increase in protein expression of the KV2.1 channel in the mPFC of depressive animal models [61]. Pharmacological treatments or loss of function have demonstrated improvements in depressive-like behaviors in these animals [62]. These findings suggest that the neurobehavioral pathophysiology associated with depression in the mPFC region is linked to abnormal potassium channel function, underscoring the critical role of potassium channels in the antidepressant process.

### Potassium channels in the hippocampal region

Damage or dysfunction of the hippocampus is also linked to depression and other emotional disorders [63]. Potassium channels in the hippocampal region are critical for neuronal activity and the development of depression. In mice, a reduction in KV3.1 levels in parvalbumin-positive (PV⁺) cells within the hippocampus leads to decreased high-frequency firing capacity of dentate gyrus PV^+^ cells, and the emergence of depressive-like behaviors. Conversely, upregulating KV3.1 or acutely activating KV3.1 with specific agonists alleviates depressive-like behaviors in mice [64]. The anesthetic ketamine exerts its sustained antidepressant effects by specifically modulating the KCNQ2 gene in glutamatergic neurons of the ventral hippocampus in mice [65]. Additionally, in lipopolysaccharide (LPS) model mice, Kir4.1 protein expression in hippocampal astrocytes is elevated, and the NLRP3 inflammasome is activated. Decreasing Kir4.1 protein levels specifically in astrocytes can decrease LPS-induced NLRP3 and calpain-1 expression and improve depressive-like behaviors in these mice [66]. Studies have shown that in depressive animal model, the activity of ATP-sensitive potassium (K-ATP) channels in the hippocampus is elevated, accompanied by increased expression of Kir6.1 and Kir6.2 subunits [67]. Administration of the N-methyl-D-aspartic(NMDA) receptor antagonist memantine inhibits K-ATP channels in the hippocampus and improves depressive-like behaviors in mice [68]. These findings suggest that Potassium channels in the hippocampus contribute to the pathogenesis of depression and may represent a promising target for treatment.

Elevated expression of TREK-1 in the hippocampus of mice subjected to chronic unpredictable mild stress (CUMS) has been documented. Targeted knockdown of TREK-1 in the hippocampal region has been shown to prevent impairment of glutamatergic synaptic transmission and ameliorate depressive-like behaviors in these mice [69]. TREK-1 knockout mice exhibit increased serotonergic neuronal activity and neurotransmission, reduced immobility time in the forced swim and tail suspension tests, and demonstrate improved outcomes in various depression-related behavioral models [70]. Moreover, the knockdown of the TASK-3 channel in monoaminergic neurons within the hippocampus leads to significant antidepressant-like effects [71]. The pivotal role of ion channels in the hippocampus underscores their substantial involvement in the pathophysiology of depression and other psychiatric conditions [72].

### Potassium channels in reward-related brain regions

The reward and anti-reward circuitry encompasses several brain regions, including the NAc, the VTA and the LHb [73]. Neuronal activity in these regions is strongly linked to the onset and treatment of depression [74, 75], with potassium channels playing a crucial role. Research has demonstrated that, in the NAc of rats, accelerated inactivation of KV1.4 channels in striatal projection neurons results in diminished reward-seeking behaviors. This effect inhibits reward motivation and contributes to the development of depressive disorders [76]. Moreover, studies using the CUMS model in mice have revealed enhanced theta-frequency long-term potentiation (tLTP) in the NAc, accompanied by reduced activity of KV4.2-type potassium currents. Direct phosphorylation at Ser-616 can mitigate the alterations in tLTP mediated by KV4.2 channels in these mice [77].

Additionally, in the social defeat model of mice, reduced activity of Kv7.4 channels in dopaminergic neurons of the VTA has been observed. Activation of Kv7.4 channels can reverse depressive-like behaviors in these mice [78]. Experimental studies have shown that in depression model rats, Kir4.1 channel activity increases in the lateral habenula (LHb) region. Furthermore, interactions between astrocytes and neurons in the LHb play a role in determining neuronal firing patterns [79], which may be relevant to the mechanism of depression. Studies have shown that activation of SK channels leads to age-dependent depressive-like behaviors and cognitive deficits. Inhibition of SK channels in dopaminergic neurons within the VTA improves depressive-like behaviors in post-stroke depression models in rats [80]. Collectively, these findings underscore the significant role of potassium channels in the reward circuitry and their involvement in both the pathogenesis and treatment of depression.

## Potassium channels as potential targets for antidepressant action

### Potassium channels participated in the pathogenesis and treatment of depression

Recent research suggests that potassium channel openers and blockers can influence the development of depression. KCNQ2/3 potassium channel openers have shown antidepressant effects. Clinical trials with the KCNQ2/3 channel opener ezogabine in patients with MDD have demonstrated significant improvements in depressive symptoms and anhedonia [95, 99]. The human studies on KCNQ channel activator ezogabine also indicate that modulating KCNQ channels can regulate neuronal excitability within the reward circuits, offering a potential target for alleviating depressive symptoms [114]. Specific inhibition of TREK-1 channel activity has also demonstrated distinct antidepressant properties [113]. Medications targeting the TREK-1 channel, such as spadin, have shown promising therapeutic effects in patients with depression [115].

Potassium channels play a crucial role in regulating immobility time in the forced swim test in mice. Blockers of potassium channels, such as TEA and charybdotoxin, significantly reduce immobility time. Conversely, openers of potassium channels, such as pinacidil, minoxidil, and cromakalim, significantly increase immobility time in mice [116]. For inward-rectifying potassium channels, the Kir4.1 channel inhibitor Lys05 induces a rapid antidepressant response in mice [117]. Inhibitors of ATP-sensitive potassium channels, such as glibenclamide, reduce the hypothalamic–pituitary–adrenal axis hyperactivity, depression, and anxiety-related symptoms in Alzheimer's disease rat models [118]. Moreover, administering the K-ATP channel opener iptakalim significantly increases sucrose preference and reduces immobility time in the forced swim test, thereby improving depressive-like behaviors in chronic mild stress-induced depression model mice [119].

The neuroprotective agent sipatrigine, which significantly inhibits the TREK-1 channel at therapeutic concentrations, exhibits notable antidepressant effects in the forced swim and other depression models [120]. Additionally, TASK-3 channel knockout mice show significantly reduced immobility time in the tail suspension and forced swim tests compared to wild-type controls. They also exhibit enhanced locomotor activity in the novel object recognition test [112]. Collectively, these studies suggest that directly influencing potassium channel activity can alleviate depressive symptoms, highlighting the critical role of potassium channels in both the pathogenesis and treatment of depression.

### Potassium ion channels can serve as therapeutic targets for antidepressant drugs

Recent studies increasingly suggest that potassium channels may play a significant role as targets for antidepressant drugs. Studies have shown that the antidepressant fluoxetine markedly enhances delayed rectifier potassium currents in cerebellar granule neurons and transient outward potassium currents in the hippocampus of hamsters [121]. Additionally, KV4.2 knockout rats display notable depressive tendencies in forced swimming and other depression tests, and fluoxetine does not reverse this depressive phenotype. This finding implies that KV4.2 might be a target of fluoxetine in the treatment of depression [90]. Fluoxetine increases the inactivation rate of KV4.3 currents in a concentration-dependent manner [122]. Various voltage-dependent potassium channel subtypes, such as KV3.1 and KV1.1, which are highly expressed in the brain, also exhibit significant inhibitory effects [123, 124]. Furthermore, fluoxetine effectively inhibits GIRK2 channels expressed in Xenopus oocytes and significantly reduces cell death in cerebellar and pontine neurons, thereby enhancing motor abilities in mice [99].

Other studies have shown that the antidepressant citalopram can decrease KV1.5 currents [84], while escitalopram inhibits TREK-1 currents in the hippocampus and prefrontal cortex of post-stroke depression model rats [125]. Chlorpromazine also treats depression and other psychiatric disorders by activating BK channels at the whole-cell level [105]. Additionally, ketamine reduces the surface density of Kir4.1 channels in astrocytes, which modulates neuronal excitability and alleviates depressive symptoms [126, 127]. These findings collectively suggest that potassium channels are potential targets for antidepressants and play a role in both the pathogenesis and treatment of depression.

## Conclusion and future perspectives

**Fig. 2:**
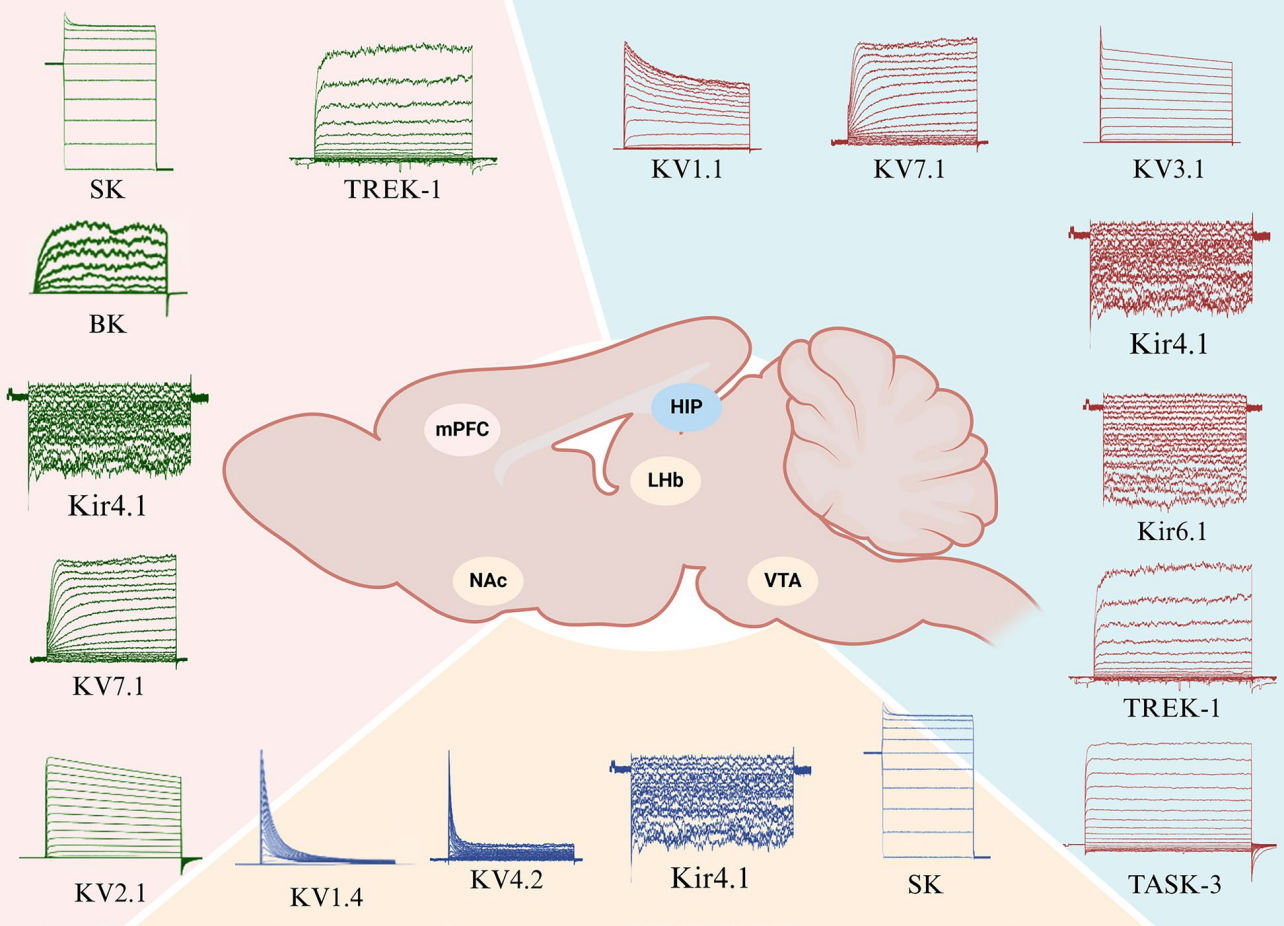
Role of various potassium channels in the pathogenesis and treatment of depression. During the onset and treatment of depression, various types of potassium channels play significant roles in different brain regions

This review delineates the role of potassium channels in depression. The available research evidence suggests multiple potassium channels have emerged as potential targets for antidepressant drugs. Meanwhile, we summarized several potassium channel gene polymorphisms associated with depression, which may have predictive value for susceptibility to depression and response to treatment. As far as is known from the existing studies, the complex relationship between potassium channels and depression involves not only the different brain areas (mPFC, hippocampal, NAc, etc.), but also the various cells(dopaminergic neurons, PV^+^ cells, astrocytes, etc.) involved, forming a sophisticated network to affect onset of depression or produce antidepressant effect (Fig. 2). But the connections between potassium channels and their relationships with depression across multiple brain regions and the specific signaling pathways involved remain unclear. Therefore, depth research into the role of potassium channels in depression is crucial. Uncovering the roles of these key channels in the pathogenesis of depression will advance our understanding and may reveal new therapeutic targets for this debilitating disorder.

## Data Availability

Not applicable.

## Abbreviations

KV: Voltage-gated K+ channels
Kir: Inward rectifier K+ channels
KCa: Calcium-activated K+ channels
K2P: Two-pore domain K⁺ channels
K-ATP: ATP-sensitive potassium
TMs: Transmembrane helices
BK: Big conductance K+ channels
IK: Intermediate conductance K+ channels
SK: Small conductance K+ channels
PFC: Prefrontal cortex
mPFC: Medial prefrontal cortex
CUMS: Chronic unpredictable mild stress
RMP: Resting membrane potential
NAc: Nucleus accumbens
VTA: Ventral tegmental area
tLTP: Theta-frequency long-term potentiation
MDD: Major depressive disorder
LHb: Lateral habenula
LPS: Lipopolysaccharide
SNPs: Single nucleotide polymorphisms
HCN: Hyperpolarization-activated cyclic nucleotide-gated
NMDA: N-methyl-d-aspartic
